# Preparation, Characterization, and Preliminary Imaging Study of [^188^Re]Re-Ibandronate as a Novel Theranostic Radiopharmaceutical for Bone Metastasis

**DOI:** 10.1155/2022/7684076

**Published:** 2022-02-25

**Authors:** Tingting Xu, Yingwei Wang, Zan Chen, Hanxiang Liu, Songsong Yang, Guangfu Liu, Yan Zhao, Wenhui Fu, Lin Liu, Ke Xiang, Dengsai Peng, Yue Chen

**Affiliations:** ^1^Department of Nuclear Medicine, The Affiliated Hospital of Southwest Medical University, Luzhou, Sichuan 646000, China; ^2^Nuclear Medicine and Molecular Imaging Key Laboratory of Sichuan Province, Luzhou, Sichuan 646000, China; ^3^Academician (Expert) Workstation of Sichuan Province, Luzhou, China; ^4^Department of Spinal Surgery, The Affiliated Hospital of Southwest Medical University, Luzhou, Sichuan 646000, China

## Abstract

**Background:**

Bone is a common site of metastasis from a malignant tumor. Several radiopharmaceuticals are available to relieve bone pain in patients with cancer. However, every radiopharmaceutical has its own disadvantages, and there is still a need to investigate easily accessible and high bone affinity radiopharmaceuticals. Ibandronate (IBA) and ^188^Re were used for radiolabeling to develop and evaluate a novel type of bone-seeking radiopharmaceutical.

**Methods:**

The preparation conditions of [^188^Re]Re-IBA were investigated, and thin-layer chromatography was used to analyze radiochemical purity. The stability, plasma protein binding rate, lipid-water distribution coefficient, safety and biodistribution in normal mice, and bone imaging of [^188^Re]Re-IBA in New Zealand rabbits were studied. In addition, the nude mice model of bone metastasis was established, and biodistribution and imaging characteristics of [^188^Re]Re-IBA in these nude mice were studied.

**Results:**

[^188^Re]Re-IBA was successfully prepared with radiochemical purity >95%. The optimum preparation conditions were as follows: IBA, 0.8–1.4 mg; ascorbic acid, 0.2–0.5 mg; stannous chloride, 0.14–0.18 mg; potassium perrhenate, 0.005 mg; and [^188^Re]ReO_4_^−^ activity, 18.5–296 MBq, reacted for 30 min at 95°C with pH = 2. [^188^Re]Re-IBA demonstrated good stability, high plasma protein binding rate, good hydrophilicity, and low toxicity. The biodistribution and bone imaging in normal animals showed rapid blood clearance, high bone uptake, low uptake in the solid organs and soft tissue, and high contrast during imaging. The biodistribution and imaging of bone metastasis in nude mice showed that [^188^Re]Re-IBA has higher uptake in bone metastasis lesions than normal bone.

**Conclusions:**

Our study encompassed the successful preparation of [^188^Re]Re-IBA, a novel bone-seeking radiopharmaceutical, and confirmed it has potential in the treatment of bone metastasis and monitoring through imaging.

## 1. Introduction

Bone is a common site of metastasis from a malignant tumor. Patients with bone metastasis usually experience severe and refractory pain. In addition, it may be accompanied by pathological fractures, spinal cord compression, hypercalcemia, and other complications, thus seriously affecting the quality of life [1]. Therefore, timely and effective treatment is needed to alleviate the symptoms and improve the quality of life.

The currently available treatments for bone metastasis are chemotherapy, external beam radiotherapy, surgery, bisphosphonate therapy, hormone therapy, and the use of painkillers [1]. In addition, the use of bone-seeking radiopharmaceuticals is also an effective method of relieving bone pain. It has the advantages of simultaneous treatment of multiple metastases, repeatability, and combination with other treatments [2]. Moreover, it can reduce or delay the incidence of skeletal-related events [3]. Numerous radiopharmaceuticals are available to relieve bone pain in patients with cancer, including [^89^Sr]SrCl_2_, [^153^Sm]Sm-ethylenediamine tetramethylene phosphonate (EDTMP), [^186^Re]Re-bisphosphonate hydroxyethylidene diphosphonate (HEDP), [^188^Re]Re-HEDP, [^177^Lu]Lu-EDTMP, and [^223^Ra]RaCl_2_ [4, 5]. However, every radiopharmaceutical has its advantages and disadvantages. The choice is extremely dependent on the status of the patient, such as the renal function, bone marrow reserve, cancer extent, and physical properties of the radionuclides [6]. As the commercial availability of radiopharmaceuticals is limited, the availability of each radiopharmaceutical also needs to be considered. Moreover, most radionuclides used for treatment are produced through reactors; thus, they are extremely expensive. Therefore, it is important to choose an easily accessible and cost-effective radiopharmaceutical. Radionuclide ^188^Re has an advantage in this regard because of its commercial extraction from ^188^W/^188^Re generators, which can be used on-demand and are cost-effective.

^188^Re has a physical half-life of 16.9 h and can produce *β*^−^ rays with a maximum energy of 2.1 MeV which can be used for treatment [7]. It also emits *γ* rays with an energy of 155 keV for imaging, which facilitates visualizing the distribution of radioactive tracers in the body during treatment [7]. [^188^Re]Re-HEDP is one of the most widely studied bisphosphonate radiopharmaceuticals in nuclear medicine which can relieve bone pain caused by prostate cancer, breast cancer, or other tumors [8]. In addition, a variety of new ^188^Re labeled bisphosphates have been synthesized, such as [^188^Re]Re-diethylenetriamine-N,N,N′,N″,N″-pentakis acid [9], [^188^Re]Re-nitrilotris acid [10], [^188^Re]Re-ethylenediamine–N,N,N,N-tetrakis acid [11], [^188^Re]Re-pamidronate (PMA) [6], [^188^Re]Re-zoledronate (ZNA) [12], and [^188^Re]Re-risedronate [13]. These drugs show potential for application in preclinical studies. It is still necessary to identify bisphosphonates with a stronger ability to target bones for ^188^Re labeling. Bisphosphonates are analogues of endogenous pyrophosphates, characterized by P-C-P bonds. There is a hydroxyl group attached to one side of the carbon atom, which has a high affinity for calcium phosphate, the primary mineral of the bone [6]. Moreover, there is a side-chain structure attached to the other side of the carbon atom that inhibits bone resorption [6]. The side-chain structure of the first-generation bisphosphonate does not contain nitrogen, and HEDP is one of its representative drugs. The second- and third-generation bisphosphonates contain nitrogen, and their ability to inhibit bone resorption is significantly stronger. Furthermore, third-generation bisphosphonates also contain a heterocyclic structure, and their bone affinity and ability to inhibit bone resorption are significantly stronger. However, nitrogen-containing bisphosphonates may have significant side effects, including renal failure, hypocalcemia, and osteonecrosis of the jaw [14]. The choice of drugs with lower toxicity is an important factor in determining the treatment for patients with renal insufficiency. Ibandronic acid and zoledronic acid are the most powerful and widely used third-generation bisphosphonates. The studies in [15, 16] on the effects of the aforementioned bisphosphonates on renal safety have reported on the possible occurrence of nephrotoxicity while using zoledronic acid. Nonetheless, the nephrotoxicity of ibandronic acid is extremely low and equivalent to that of the placebo. Another study [17] conducted on 44 patients treated with ibandronate (IBA) reported no impairment of renal function during an average follow-up of 18.5 months. In addition, Han et al. [18] noted that the pain relief rate and improvement in the quality of life in patients with bone tumors were higher in the ibandronic acid group than in the zoledronic acid group (*P* < 0.05). Nonetheless, the rate of adverse reactions was lower in the ibandronic acid group than in the zoledronic acid group (*P* < 0.05). There have been no reports on ^188^Re labeled with IBA. Therefore, IBA and ^188^Re were selected for radiolabeling to develop and evaluate a new radiopharmaceutical with potential bone-seeking properties and low toxicity, which may contribute to individualized treatment in the era of precision medicine. Light will be shed on the preparation, optimization of conditions, biological and safety evaluation, and imaging studies of [^188^Re]Re-IBA.

## 2. Materials and Methods

### 2.1. Materials

[^188^Re]NaReO_4_ was eluted from the alumina based ^188^W/^188^Re generator (OncoBeta, Germany) with saline solution (0.9% NaCl). IBA was purchased from Twbio Technology Co., Ltd., Beijing, China. Ascorbic acid, potassium perrhenate (KReO_4_), and stannous chloride (SnCl_2_) were purchased from Macklin Biochemical Co., Ltd., Shanghai, China. Xinhua No. 1 chromatography paper (Xinhua Paper Industry Co., Ltd., Hangzhou, China) was used for paper chromatography (PC). Other equipment, chemicals, and animals used in the experiment were provided by the Nuclear Medicine and Molecular Imaging Key Laboratory of Sichuan Province. All studies were approved by the Ethics Committee of Southwest Medical University.

### 2.2. Methods

#### 2.2.1. Radiolabeling and Quality Control

**Figure 1:**
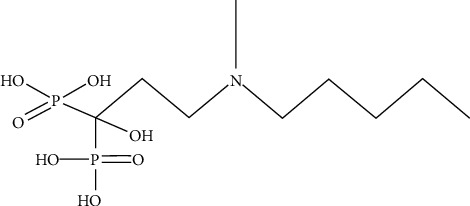
Chemical structure of the IBA.

Various reaction parameters were studied by using the control variables method (changing one parameter at a time) to determine the effects of IBA (Figure 1), the antioxidant (ascorbic acid), carrier (KReO_4_), reducing agent (SnCl_2_), [^188^Re]ReO_4_^−^ activity, pH, temperature, and reaction time on the radiochemical purity (RCP) of [^188^Re]Re-IBA. First, 0.1–1.8 mg, 0–0.5 mg, 0.02–0.4 mg, and 0–0.019 mg of IBA, ascorbic acid, SnCl_2_, and KReO_4_ were mixed sequentially. Then, the fresh eluted [^188^Re]ReO_4_^−^ solution 18.5–444 MBq was added. Subsequently, the pH value was adjusted to 0.5–4 with 1 N sodium acetate solution and 1 N hydrochloric acid. The reaction occurred at room temperature (25 ± 2°C), 60°C, or 95°C for 10–60 min, respectively. After the reaction was completed, the solution was cooled to room temperature (25 ± 2°C). The pH value of each tube was adjusted to 6–7. An aseptic filter membrane of 0.22 *μ*m was used for sterilization and filtration. In this study, the specific fixed values of all parameters were as follows: IBA, 1.0 mg; ascorbic acid, 0.3 mg; SnCl_2_, 0.14 mg; KReO_4_, 0.005 mg; and [^188^Re]ReO_4_^−^ activity, 37 MBq, reacted for 30 min at 95°C and pH = 2.

The RCP of [^188^Re]Re-IBA was determined by PC. Acetone and 0.9% NaCl were used as the eluents. A TLC scanner was used to measure the distribution of radioactivity on the PC strips. The RCP of [^188^Re]Re-IBA was calculated from the peak area measurements as follows:

RCP = 100% − (% [^188^Re]ReO_4_^−^ + % [^188^Re]Re-colloid).

#### 2.2.2. In Vitro Stability

Freshly prepared [^188^Re]Re-IBA was incubated in 0.9% NaCl and fresh human serum at 37°C. The RCP of the tubes was determined by PC at 1 h, 3 h, 6 h, 12 h, and 24 h. The experiment was repeated thrice. The results are expressed as mean ± standard deviation ($\bar{\chi }$ ± *s*).

#### 2.2.3. Plasma Protein Binding Rate

0.1 mL fresh human plasma and freshly prepared 1.85 MBq [^188^Re]Re-IBA were added in a tube labeled A and incubated at 37°C for 2 h. Then, 25% trichloroacetic acid solution (1 mL) was added to tube A and centrifuged under a centrifugal force of 587 g for 5 min. The supernatant was collected into another tube labeled B. Centrifugation was repeated, and the supernatant was collected thrice. A *γ* counter was used to measure the radioactivity counts of tubes A and B. After repeating the experiment thrice, the plasma protein binding (PPB) rate was calculated as follows: PPB = [(*A* − background)/(*A* + *B* − background × 2)] × 100%. The results are expressed as mean ± standard deviation ($\bar{\chi }$ ± *s*).

#### 2.2.4. Lipids and Water Distribution Coefficient

Freshly prepared 1.85 MBq [^188^Re]Re-IBA was added to a tube labeled A and it was shaken for 20 min with a vortex mixer, followed by centrifugation at 587 g for 5 min. The 0.1 mL upper liquid (organic phase) was collected into a tube labeled B. The 0.1 mL lower liquid (water phase) was collected into a tube labeled C. The radioactivity counts of the organic phase and water phase were measured by a *γ* counter. After repeating the experiment thrice, the lipid-water partition coefficient (log*P*) was calculated by using the following formula: log*P* = log[(B-background)/(C-background)]. The results are expressed as mean ± standard deviation ($\bar{\chi }$ ± *s*).

#### 2.2.5. Toxicity Test of Mice

Sixteen Kunming mice were randomly divided into four groups (equal number of males and females): normal control group and low-, middle-, and high-dose [^188^Re]Re-IBA groups. The control group was injected with 0.9% NaCl, and the experimental groups were injected with [^188^Re]Re-IBA solution of 3.7 MBq, 18.5 MBq, and 37 MBq, respectively. The body weight and general condition of the mice in each group were measured. After 4 weeks, the blood of the mice in each group was drawn for routine blood examination and liver and kidney function. A pathological examination of important tissue and organs was performed.

#### 2.2.6. The Biodistribution of [^188^Re]Re-IBA and [^188^Re]ReO_4_^−^ in Mice

Twenty Kunming mice (originated from Swiss albino mice and introduced into Kunming, China.) were randomly divided into five groups (equal number of males and females). Each group was injected with 3.7 MBq [^188^Re]Re-IBA. Then, the mice were sacrificed by CO_2_ asphyxiation at 1 h, 3 h, 6 h, 24 h, and 48 h. The blood and important tissue and organ samples were collected to measure the radioactivity count by *γ* counter. Following the time attenuation correction, the percentage injection dose rate per gram of tissue (%ID/g) was calculated at each time point. The result is expressed as mean ± standard deviation ($\bar{\chi }$ ± *s*). The in vivo distribution of [^188^Re]ReO_4_^−^ was studied using the aforementioned method.

#### 2.2.7. Imaging of New Zealand Rabbits with [^188^Re]Re-IBA

The New Zealand rabbit was anesthetized by intraperitoneal injection of 2% pentobarbital sodium (40 mg/kg). Then, 100 MBq [^188^Re]Re-IBA was injected. Bone imaging was performed at different time points (acquisition equipment: American GE Infinia T4 double probe single-photon emission computed tomography, high energy collimator; scanning parameters: energy peak: 155 keV, posture: supine position, matrix: 128 × 128, and window width: ±10%). Following image acquisition, the images were processed by using the software of the postprocessing workstation.

#### 2.2.8. Biodistribution and Imaging of Bone Metastasis Nude Mice

The nude mice of the bone metastasis model were established by tibial bone marrow injection. The specific methods were as follows: 25 *μ*L of prostate cancer PC-3 and breast cancer MDA-MB-231 cell culture medium were injected into the left tibial bone marrow cavity of healthy male and female nude mice, respectively. At approximately 3-4 weeks after inoculation, they were scanned by microcomputed tomography (CT, SIEMENS InveonTM, Munich, Germany) to determine the condition of the model. If CT showed bone destruction in the left tibia (osteolytic, osteogenic, or mixed types); it suggested that the model was successful. Bone metastases were initially evaluated by CT and confirmed by pathology after the completion of related studies.

Ten PC-3 and ten MDA-MB-231 nude mice with bone metastasis were randomly divided into five groups. The mice were sacrificed by CO_2_ asphyxiation at 1 h, 3 h, 6 h, 24 h, and 48 h. The important tissue and organ samples were collected for the biodistribution study.

The nude mice were injected with [^18^F]F-sodium fluoride (NaF), [^99m^Tc]Tc-methylene diphosphonate (MDP), and [^188^Re]Re-IBA at the first, second, and fifth days, respectively, for bone imaging. Photon emission tomography/computed tomography (PET/CT) acquisition equipment was SIEMENS InveonTM. Single-photon emission computed tomography/computed tomography (SPECT/CT) acquisition equipment was GE SPECT, pinhole collimator.

#### 2.2.9. Statistical Analysis

SPSS 26.0 was used for statistical analysis, and the quantitative data were expressed as mean ± standard deviation ($\bar{\chi }$ ± *s*). In the toxicity test, repeated-measures analysis of variance was used to compare the body weight of mice in each group. *P* < 0.05 was considered statistically significant.

## 3. Results

### 3.1. Radiolabeling and Quality Control

**Figure 2:**
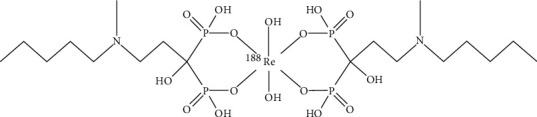
The proposed structure of [^188^Re]Re-IBA.

**Figure 3:**
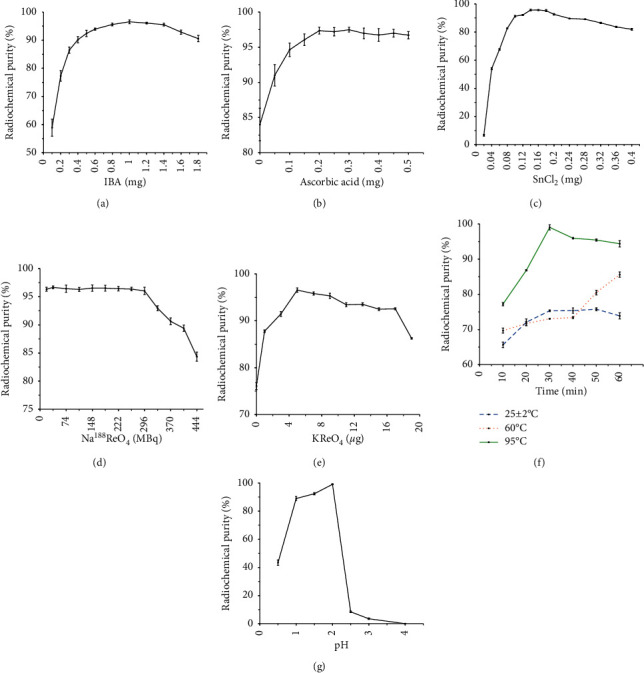
Effects of IBA (a), ascorbic acid (b), SnCl_2_ (c), [^188^Re]ReO_4_^−^ activity (d), KReO_4_ (e), temperature and reaction time (f), and pH (g) on the radiochemical yield of [^188^Re]Re-IBA.

**Figure 4:**
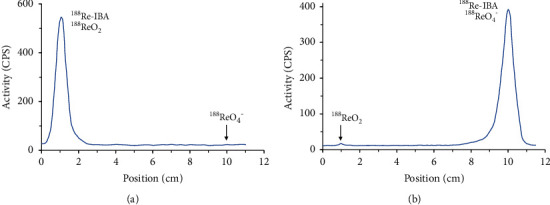
Typical PC distribution of [^188^Re]Re-IBA in acetone (a) and saline (b).

A third-generation bisphosphonate derivative targeting bone metastasis [^188^Re]Re-IBA was prepared. The proposed structure of [^188^Re]Re-IBA is represented in Figure 2. The optimum preparation conditions in our study were as follows (see Figure 3): IBA, 0.8–1.4 mg; ascorbic acid, 0.2–0.5 mg; SnCl_2_, 0.14–0.18 mg; [^188^Re]ReO_4_^−^ activity, 18.5–296 MBq; and KReO_4_, 0.005 mg, reacted for 30 min at 95°C and pH = 2. Typical radioactivity distribution in PC is shown in Figure 4. Upon using acetone as an eluent, the colloidal impurities [^188^Re]ReO_2_ and product [^188^Re]Re-IBA remained at the origin of the PC strip; however, [^188^Re]ReO_4_^−^ moves with the eluent front (Figure 4(a)). In contrast, upon using saline as the eluent, the colloidal impurities [^188^Re]ReO_2_ remained at the origin, while [^188^Re]ReO_4_^−^ and the product [^188^Re]Re-IBA move with the eluent front (Figure 4(b)). [^188^Re]Re-IBA could be consistently prepared in > 95% RCP under the best preparation conditions. Moreover, it did not require further purification.

### 3.2. In Vitro Stability

Table 1 summarizes the results of in vitro stability. The results show that RCP is 91.44% and 93.06% in 0.9% NaCl and serum at 24 h, respectively, showing that [^188^Re]Re-IBA has good stability.

### 3.3. Plasma Protein Binding Rate and Lipids and Water Distribution Coefficient

The PPB of [^188^Re]Re-IBA incubated in plasma for 2 h was 79.8 ± 0.71%. log*P* of [^188^Re]Re-IBA was - 2.33 ± 0.02, which indicated its high hydrophilicity.

### 3.4. Toxicity Test of Mice

**Figure 5:**
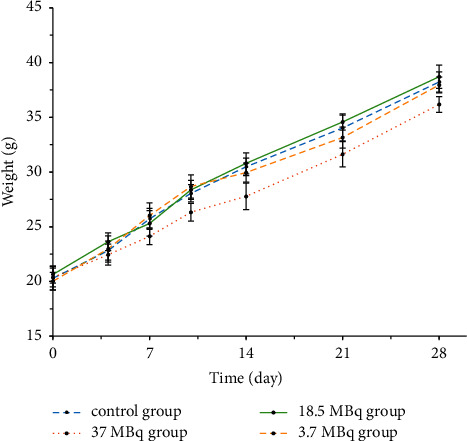
Weight change of mice injected with normal saline or different doses of [^188^Re]Re-IBA within 4 weeks.

**Figure 6:**
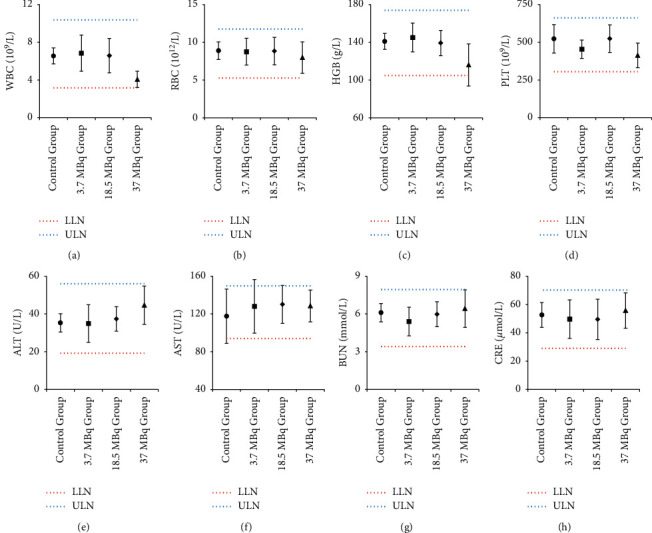
Routine blood (WBC, RBC, HGB, and PLT), liver function (ALT and AST), and renal function (blood urea nitrogen and serum creatinine) of normal mice at 4 weeks after injection of normal saline or different doses of [^188^Re]Re-IBA. The results are expressed as mean ± standard deviation ($\bar{\chi }$ ± (s) (LLN: lower limit of normal; ULN: upper limit of normal).

**Figure 7:**
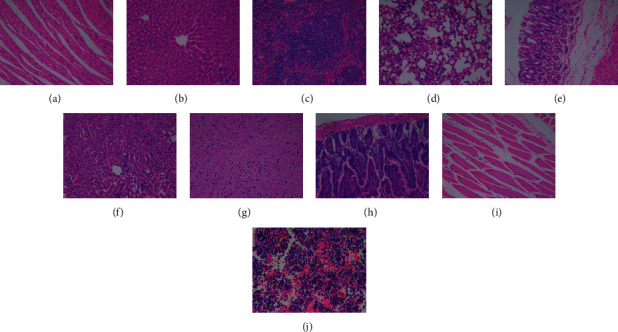
Pathology of mice at 4 weeks after injection of 0.9% NaCl (normal control group): (a) heart; (b) liver; (c) spleen; (d) lung; (e) stomach; (f) kidney; (g) brain; (h) small intestine; (i) muscle; (j) bone marrow. (a)–(i) HE × 200; (j) HE × 400.

**Figure 8:**
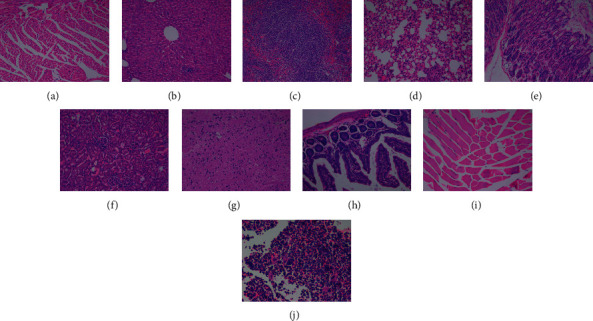
Pathology of mice at 4 weeks after injection of 37 MBq [^188^Re]Re-IBA (high-dose group): (a) heart; (b) liver; (c) spleen; (d) lung; (e) stomach; (f) kidney; (g) brain; (h) small intestine; (i) muscle; (j) bone marrow. (a)–(i) HE × 200; (j) HE × 400.

There were no adverse reactions in all mice within 4 weeks, and the weight of each group increased with the increase of time (see Figure 5). The mean values of routine blood and liver and kidney function examination of the four groups were within the normal reference range (see Figure 6). Compared with the normal control group (see Figure 7), there were no abnormal pathological changes in important tissues and organs among the low-dose group (3.7 MBq), medium-dose group (18.5 MBq), and high-dose group (see Figure 8). In addition, there was no significant difference in weight among the low-dose group (3.7 MBq), medium-dose group (18.5 MBq), and control group. However, there were significant differences between the high-dose group (37 MBq) and the other three groups, and the average difference between the high-dose group and the other three groups was negative, which suggested that high-dose [^188^Re]Re-IBA inhibited the weight gain of mice to a certain extent. However, the dose of 37 MBq was too high for mice, and the body weight of this group still showed an increasing trend, and the pathological results were not abnormal, suggesting that [^188^Re]Re-IBA is safe and has low toxicity.

### 3.5. The Biodistribution of [^188^Re]Re-IBA and [^188^Re]ReO_4_^−^ in Mice and Imaging of New Zealand Rabbits with [^188^Re]Re-IBA

Table 2 summarizes the biodistribution of [^188^Re]Re-IBA in mice. A rapid blood clearance and low uptake of [^188^Re]Re-IBA in the soft tissue, brain, liver, lung, and spleen were observed. The bone uptake of [^188^Re]Re-IBA is relatively high, and the %ID/g reaches the maximum at 6 h (7.724 ± 2.292%ID/g), and %ID/g remains 5.239 ± 2.029 at 48 h. In addition, the radioactivity ratio of the bone to heart, liver, and muscles is rather high at 48 h. In addition to the bone, the highest uptake of [^188^Re]Re-IBA occurred in the kidneys, which is related to the kidney as the primary excretory organ.  Table 3 outlines the distribution of [^188^Re]ReO_4_^−^ in mice. The highest uptake of [^188^Re]ReO_4_^−^ occurs in the stomach and thyroid gland. However, the uptake in the bone is negligibly low. Furthermore, the radioactivity ratio of bone to heart, liver, and muscle tissue is low, and the highest ratio is as low as 2.372 (bone/muscle, 3 h).

**Figure 9:**
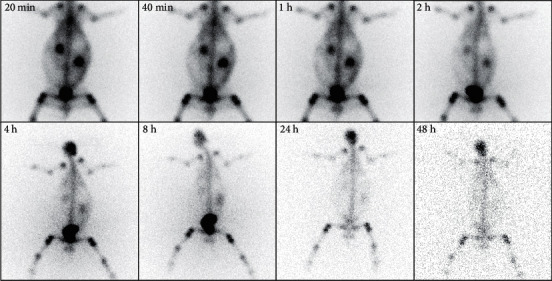
Bone imaging of New Zealand rabbits injected with [^188^Re]Re-IBA at 20 min–48 h.

Figure 9 depicts the bone imaging of the New Zealand rabbits in different time points. There was an obvious accumulation in bones and mild tracer uptake in soft tissue at 20 min after the injection. Over time, the soft tissue accumulation gradually faded and disappeared, and the bone imaging was clear with a high contrast between the bone and the background. In addition, the elimination of most of the [^188^Re]Re-IBA activity was through the kidneys, which was consistent with the biodistribution in mice.

### 3.6. Biodistribution and Imaging of Bone Metastasis Nude Mice

Table 4 summarizes the biodistribution of [^188^Re]Re-IBA in bone metastasis nude mice. A rapid blood clearance and low uptake in soft tissue, brain, liver, lung, and spleen of [^188^Re]Re-IBA were observed, which is consistent with the biodistribution in mice. The comparison between the model bone and the contralateral bone shows that the uptake of the model bone is lower than that of the contralateral bone at 1–3 h but higher than that of the contralateral bone from 6 to 48 h.

[^188^Re]Re-IBA bone imaging of nude mice with bone metastasis showed that, at 1–3 h after injection, the uptake in the model side was higher than that of the control side, but the concentration was mainly distributed in the surrounding soft tissue; at 6 h, 16 h, and 32 h, the bone uptake of the model bone was higher than that of the contralateral bone.  Table 5 shows the ROI ratio of model bone/contralateral bone in nude mice of bone metastasis model at 6 h, 16 h, and 32 h.

## 4. Discussion

The amount of ligand IBA is an important evaluation parameter in the labeling process. A sufficient number of ligands must be provided in the formula to achieve high RCP complex formation. However, using excessive quantities of bisphosphonate ligand should be avoided to achieve high specific activity labeling and avoid the formation of undesired side product, which increases the impurity and inversely affects the labeling yield [6, 12]. Our study found that the highest RCP complex formation can be obtained when the number of ligands in the formula is 0.8–1.4 mg. With further increase of the dosage, the RCP gradually decreased, which may be due to the nonlabeled ligand which interfered with the labeling process or the presence of too many ligands making the reaction solution supersaturated and thus affecting the labeling. In this study, ascorbic acid was used as the antioxidant and stannous ion stabilizer. The results showed that when the quantity of ascorbic acid in the formula was 0.15–0.5 mg, [^188^Re]Re-IBA could be prepared in >95% RCP. An inorganic compound of tin, SnCl_2_, has been widely used in nuclear medicine as a reducing agent for pharmaceutical products radiolabeled with ^99m^Tc or ^188^Re [19]. In our study, SnCl_2_ was chosen as the reducing agent to reduce [^188^Re]ReO_4_^−^ to a lower oxidation state and facilitate its reaction with IBA. Despite its widespread use, there are several side effects associated with this ion and its derivatives described in the literature, for example, irritation of oral mucosa in rats [20], deterioration in semen quality of male rabbits [21], free radical induction and damage to liver and kidney function in rabbits [22], and marked hazardous alterations in some enzymatic activities and biochemical parameters in rabbits [23]. Furthermore, genotoxicity, mainly related to SnCl_2_, has been reported [24], which could mediate single-strand breaks in plasmid DNA through reactive oxygen species (ROS) formation [19]. Our research showed that the RCP is high enough when the amount of SnCl_2_ in the formula reaches 0.14–0.18 mg. An increase in the amount of SnCl_2_ beyond this dosage range is not beneficial to the labeling yield and might lead to the formation of colloidal impurities in the product. However, an insufficient amount of SnCl_2_ will not reduce all [^188^Re]ReO_4_^−^. Therefore, it is necessary to ensure an optimum ratio of SnCl_2_ with IBA and [^188^Re]ReO_4_^−^, and the excess SnCl_2_ should be removed as much as possible. In addition, avoiding the potential toxicity of SnCl_2_ in the formula is also a problem worth discussing. Several studies showed that ascorbic acid, a well-known antioxidant and water-soluble ROS scavenger, is capable of detoxifying the hazardous effects of SnCl_2_, including alleviation of reproductive toxicity [21] and the toxicity to some enzyme activities and oxidative damage [22], as well as increase in the activities of antioxidant enzymes [23]. Therefore, the ascorbic acid used in our formula may also act as a protectant against the toxicity of SnCl_2_. The ^188^Re obtained in the generator was carrier-free. In previous studies [9, 25–32], [^188^Re]Re-bisphosphonate, without cold carrier rhenium, had a poor stability and little bone uptake. Hence, nonradioactive KReO_4_ was added as a cold perrhenate carrier to ensure the stability and bone uptake.

In addition, [^188^Re]Re-IBA, like [^188^Re]Re-HEDP [33], [^188^Re]Re-ZNA [12], and [^188^Re]Re-ABP [34], may be an anionic six-coordinated complex with one metal atom bound to two IBA ligand molecules. Figure 2 shows the proposed structure of [^188^Re]Re-IBA. However, the structure we provide is only a basic coordination structure. In fact, [^188^Re] Re-IBA is likely to exist in a form with a complex spatial structure. Elder et al. [35] carefully studied the structure of Re-HEDP and found that medically effective species of Re-HEDP with Re-Re bonds may exist in two forms of complex mixture: as a linear tetramer of rhenium atoms bridged by HEDP ligands, HEDP ligands which also bind an equivalent number of tin atoms with additional HEDP ligands, or as a triangular cluster of rhenium atoms bicapped by two HEDP ligands and bridged to three tin atoms by HEDP to form a complex. As a similar compound of Re-HEDP, we speculate that [^188^Re]Re-IBA may also exist in a similar structural form. Therefore, its structure may contain Re-Re bonds and tin atoms, bridged by HEDP ligands, forming a complex spatial structure. However, at present, little is known about the specific structure of this radiopharmaceutical. To determine the exact structure of [^188^Re]Re-IBA, further research should be pursued.

The stability of radiopharmaceuticals is extremely important for therapy. Our study showed that the [^188^Re]Re-IBA has good stability, which is beneficial for further research. The differences in stability between [^188^Re]Re-IBA and other drugs were also compared, and the results showed that there was no significant difference in stability between [^188^Re]Re-IBA and [^188^Re]Re-HEDP (91.44 ± 0.8% versus 91.2% at 24 h) in 0.9% NaCl [32], but its stability improved compared to [^188^Re]Re-risedronate (80.3% at 24 h) and [^188^Re]Re-PMA (81.9% at 24 h) [6, 13]. In addition, the stability of [^188^Re]Re-IBA (93.06 ± 1.6% at 24 h) in human serum was improved compared to those of [^188^Re]Re-ZNA (91.53 ± 1.39% at 24 h)) and [^188^Re]Re-risedronate (73 ± 1.21% at 24 h) [12, 13].

Radiopharmaceuticals should achieve ideal therapeutic effects and minimize the related toxicity to gain efficacy as therapeutic drugs [4]. Toxicity test of mice showed that [^188^Re]Re-IBA is safe and has low toxicity; thus it can be used for later imaging and therapeutic research. The biodistribution and imaging of normal animals indicated that [^188^Re]Re-IBA highly targets the bone and can remain in the bone for a long time. As expected, the in vivo distribution of [^188^Re]ReO_4_^−^ revealed that the free [^188^Re]ReO_4_^−^ was poorly targeted to the bone, thus confirming the success and bone-targeting capability of the labeled [^188^Re]Re-IBA. In addition, the imaging results of the New Zealand rabbits revealed fast soft tissue clearance and low nontarget tissue uptake, and the overall image quality was good. Hence, [^188^Re]Re-IBA has potential for the treatment of bone pain and can also be used to monitor therapeutic imaging.

The bone uptake of [^188^Re]Re-IBA in normal mice was also compared with those of other ^188^Re labeled bisphosphonates [6, 12, 36–38]. Although the mouse strain used for evaluation of [^188^Re]Re-IBA (Kunming mice) is not completely the same as those used for evaluation of other ^188^Re labeled bisphosphonates (Swiss mice or Kunming mice), we believe that these data can be compared considering that Kunming mice also originate from the Swiss mice strain. We noted that, among the ^188^Re labeled third-generation bisphosphonates (i.e., IBA and ZNA), [^188^Re]Re-IBA had the highest bone uptake. The highest femur bone uptake value of [^188^Re]Re-IBA in Kunming mice was 7.724 ± 2.292%ID/g (6 h), and the minimum value was 5.239 ± 2.029%ID/g (48 h). The bone uptake of [^188^Re]Re-ZNA in Swiss mice reached the maximum at 1 h (1.08 ± 0.14%ID/g); however, with the extension of time, the bone uptake decreased gradually and reached the lowest value of 0.36 ± 0.06%ID/g at 24 h [12]. In addition, the highest and lowest bone uptakes of ^188^Re labeled second-generation bisphosphonate PMA in Swiss mice were 0.81 ± 0.25%ID/g (4 h) and 0.40 ± 0.05%ID/g (1 h), respectively [6]. Therefore, according to the existing research results, their bone activity uptake is categorized as [^188^Re]Re-IBA > [^188^Re]Re-ZNA > [^188^Re]Re-PMA. In addition, the comparison results in bone uptake of [^188^Re]Re-HEDP (Kunming mice or Swiss mice) and [^188^Re]Re-IBA (Kunming mice) showed different conclusions. In two studies [37, 38], it was found that the bone uptake of [^188^Re]Re-HEDP in Kunming mice (22.36 ± 4.11%ID/g at 24 h and 11.08 ± 4.25%ID/g at 24 h, respectively) was significantly higher than that of [^188^Re]Re-IBA (6.177 ± 2.163%ID/g at 24 h). However, in another study [36], the maximum bone uptake of [^188^Re]Re-HEDP (1.0 ± 0.1%ID/g at 4 h) in Swiss mice was significantly lower than that of [^188^Re]Re-IBA (7.724 ± 2.292%ID/g at 6 h). The differences in these results may be related to the differences in the drugs obtained by different labeling methods or the differences in experimental operation methods. The differences of osteophilic properties between [^188^Re]Re-IBA and other ^188^Re labeled bisphosphonates, especially [^188^Re]Re-HEDP, should be directly compared in future studies.

In addition, we noticed that there was a significant uptake of [^188^Re]Re-IBA in the joints of the New Zealand rabbits upon bone imaging. El-Mabhouh and Mercer [39] explained that the joints are areas of increased osteoblastic activity in young animals due to bone growth; in addition, the distribution of bisphosphonates in the bone is not uniform but will rather be concentrated in the joints where bone growth and consequently osteoblastic activity are more pronounced. This may explain the significant imaging agent activity in the joint area of the New Zealand rabbits. The joint bone surface will provide an animal model for the activity present at sites of osteoblastic bone metastases [39].

Biodistribution and imaging of bone metastasis in nude mice showed that the uptake of [^188^Re]Re-IBA in the model bone is higher than that in the normal bone, suggesting that [^188^Re]Re-IBA is more targeted in bone metastasis. However, there was only a slightly increased uptake of [^188^Re]Re-IBA in the model bone compared to that in the contralateral bone, which may be due to almost all model bone being osteolytic lesions, which may be related to the way of modelling. In addition, we speculate that [^188^Re]Re-IBA may have better bone targeting in bone metastasis than the conventional bone imaging using [^99m^Tc]Tc-MDP. However, the current research is limited by the detection performance of our SPECT/CT equipment for nude mice and the lack of an osteogenic metastasis model, which will be further improved in future research.

## 5. Conclusions

This study encompassed the successful preparation of [^188^Re]Re-IBA, a novel bisphosphonate radiopharmaceutical. The aforementioned radiopharmaceutical has the advantages of a simple preparation method, high stability and PPB, good hydrophilicity, and low toxicity. The in vivo biological distribution in mice and imaging of New Zealand rabbits confirmed the following: rapid blood clearance, high affinity to the bone, long retention time in the bone, low uptake in the solid organs and soft tissue, and high contrast of imaging. The biodistribution and imaging of bone metastasis nude mice confirmed that [^188^Re]Re-IBA has a higher bone affinity to bone metastasis lesion than normal bone. Therefore, [^188^Re]Re-IBA is a suitable candidate for the treatment of bone metastasis and monitoring the therapeutic effects. However, the method of modelling should be further improved to obtain an osteogenic metastasis model that is more suitable for research. In addition, the efficacy of [^188^Re]Re-IBA in the treatment of animal models of bone metastasis and comparisons with other radiopharmaceuticals should be explored to completely clarify its value. This is the goal of our future research.

## Figures and Tables

**Table 1:** The in vitro stability of [^188^Re]Re-IBA under different methods.

Method	1 h (%)	3 h (%)	6 h (%)	12 h (%)	24 h (%)
0.9% NaCl	98.14 ± 0.2	97.82 ± 0.6	96.99 ± 1.3	95.33 ± 1.1	91.44 ± 0.8
Serum	99.01 ± 0.5	98.67 ± 0.9	98.15 ± 0.8	96.90 ± 1.4	93.06 ± 1.6

**Table 2:** The biodistribution of [^188^Re]Re-IBA in mice (*n* = 4).

Tissue	%ID/g (%)				
	1 h	3 h	6 h	24 h	48 h
Heart	0.488 ± 0.179	0.293 ± 0.118	0.199 ± 0.049	0.045 ± 0.012	0.019 ± 0.010
Liver	1.100 ± 0.165	0.794 ± 0.133	0.526 ± 0.087	0.122 ± 0.042	0.038 ± 0.020
Spleen	0.952 ± 0.336	0.475 ± 0.135	0.296 ± 0.108	0.189 ± 0.052	0.046 ± 0.009
Lung	1.297 ± 0.661	0.784 ± 0.196	0.506 ± 0.104	0.138 ± 0.037	0.040 ± 0.020
Kidney	5.894 ± 1.648	3.140 ± 0.799	2.763 ± 0.731	0.913 ± 0.173	0.399 ± 0.153
Stomach	2.928 ± 0.851	2.801 ± 0.560	1.741 ± 0.368	0.098 ± 0.036	0.047 ± 0.019
Thyroid gland	0.594 ± 0.201	0.449 ± 0.171	0.399 ± 0.082	0.115 ± 0.036	0.070 ± 0.019
Small intestine	0.898 ± 0.494	0.597 ± 0.214	0.608 ± 0.192	0.078 ± 0.013	0.033 ± 0.010
Blood	1.869 ± 0.665	0.785 ± 0.220	0.496 ± 0.207	0.058 ± 0.013	0.031 ± 0.009
Brain	0.072 ± 0.023	0.056 ± 0.010	0.045 ± 0.011	0.024 ± 0.013	0.009 ± 0.002
Femur	5.989 ± 1.680	7.105 ± 1.901	7.724 ± 2.292	6.177 ± 2.163	5.239 ± 2.029
Muscle	0.550 ± 0.251	0.344 ± 0.143	0.220 ± 0.124	0.051 ± 0.020	0.016 ± 0.005
Gonad	0.878 ± 0.426	0.611 ± 0.095	0.303 ± 0.141	0.059 ± 0.032	0.029 ± 0.009
Femur/heart	12.278	24.214	38.835	137.083	270.814
Femur/liver	5.443	8.949	14.694	50.645	136.914
Femur/muscle	10.880	20.664	35.182	121.910	325.483

The percentage injection dose rate per gram of tissue (%ID/g) is expressed as mean ± standard deviation ($\bar{\chi }$ ± *s*).

**Table 3:** The biodistribution of [^188^Re]ReO_4_^−^ in mice (*n* = 4).

Tissue	%ID/g (%)				
	1 h	3 h	6 h	24 h	48 h
Heart	1.422 ± 0.339	0.401 ± 0.050	0.176 ± 0.074	0.017 ± 0.005	0.011 ± 0.004
Liver	2.480 ± 0.556	0.530 ± 0.038	0.258 ± 0.086	0.023 ± 0.010	0.018 ± 0.010
Spleen	2.341 ± 0.973	0.490 ± 0.090	0.270 ± 0.098	0.028 ± 0.007	0.022 ± 0.006
Lung	4.458 ± 1.379	1.026 ± 0.312	0.487 ± 0.132	0.054 ± 0.017	0.036 ± 0.008
Kidney	2.923 ± 0.658	0.683 ± 0.118	0.338 ± 0.191	0.017 ± 0.003	0.017 ± 0.007
Stomach	22.747 ± 5.67	8.578 ± 3.928	2.828 ± 1.287	0.053 ± 0.032	0.029 ± 0.012
Thyroid gland	20.247 ± 2.489	10.167 ± 1.618	4.299 ± 1.015	0.051 ± 0.026	0.025 ± 0.009
Small intestine	1.671 ± 0.448	0.558 ± 0.207	0.263 ± 0.050	0.023 ± 0.004	0.014 ± 0.005
Blood	4.648 ± 1.631	1.578 ± 0.419	0.533 ± 0.254	0.023 ± 0.009	0.007 ± 0.002
Brain	0.207 ± 0.044	0.070 ± 0.024	0.044 ± 0.019	0.017 ± 0.004	0.013 ± 0.006
Femur	1.670 ± 0.345	0.431 ± 0.038	0.213 ± 0.102	0.031 ± 0.003	0.011 ± 0.005
Muscle	0.775 ± 0.074	0.182 ± 0.023	0.109 ± 0.065	0.021 ± 0.008	0.020 ± 0.005
Gonad	1.096 ± 0.007	0.404 ± 0.130	0.207 ± 0.065	0.021 ± 0.009	0.007 ± 0.001
Femur/heart	1.195	1.075	1.205	1.853	1.060
Femur/liver	0.685	0.813	0.823	1.325	0.638
Femur/muscle	2.193	2.372	1.950	1.498	0.585

The percentage injection dose rate per gram of tissue (%ID/g) is expressed as mean ± standard deviation ($\bar{\chi }$ ± *s*).

**Table 4:** The biodistribution of [^188^Re]Re-IBA in nude mice of bone metastasis model (*n* = 4).

Tissue	%ID/g (%)				
	1 h	3 h	6 h	24 h	48 h
Heart	0.595 ± 0.099	0.326 ± 0.175	0.183 ± 0.097	0.052 ± 0.027	0.035 ± 0.013
Liver	1.236 ± 0.219	0.431 ± 0.312	0.368 ± 0.054	0.089 ± 0.027	0.044 ± 0.013
Spleen	0.530 ± 0.124	0.227 ± 0.141	0.176 ± 0.029	0.066 ± 0.024	0.022 ± 0.013
Lung	1.135 ± 0.209	0.298 ± 0.250	0.257 ± 0.030	0.101 ± 0.038	0.047 ± 0.005
Kidney	6.004 ± 0.523	2.591 ± 1.120	2.587 ± 0.126	0.640 ± 0.175	0.121 ± 0.034
Stomach	3.056 ± 0.321	2.064 ± 0.207	1.176 ± 0.190	0.192 ± 0.092	0.027 ± 0.019
Thyroid gland	0.625 ± 0.309	0.282 ± 0.149	0.354 ± 0.217	0.082 ± 0.030	0.020 ± 0.010
Small intestine	0.718 ± 0.073	0.404 ± 0.047	0.242 ± 0.118	0.049 ± 0.003	0.030 ± 0.018
Blood	1.704 ± 0.348	0.531 ± 0.092	0.302 ± 0.092	0.045 ± 0.020	0.021 ± 0.008
Brain	0.098 ± 0.046	0.082 ± 0.017	0.042 ± 0.042	0.037 ± 0.020	0.021 ± 0.009
CB	9.331 ± 0.541	7.717 ± 4.349	7.662 ± 2.934	4.413 ± 2.225	4.737 ± 0.863
MB	8.329 ± 0.329	5.922 ± 2.126	8.417 ± 1.820	6.403 ± 0.247	6.503 ± 0.010
CS	0.578 ± 0.043	0.524 ± 0.172	0.153 ± 0.083	0.081 ± 0.050	0.041 ± 0.022
Gonad	0.439 ± 0.162	0.231 ± 0.068	0.106 ± 0.022	0.069 ± 0.018	0.028 ± 0.001
CB/heart	15.682	23.637	41.876	85.157	133.599
CB/liver	7.549	17.896	20.818	49.848	106.617
CB/CS	16.144	14.731	50.044	54.620	115.161
MB/heart	14.003	18.139	45.999	123.563	183.399
MB/liver	6.738	13.734	22.867	72.330	146.359
MB/CS	14.411	11.305	54.970	79.255	158.088
MB/CB	0.893	0.767	1.099	1.451	1.373

The percentage injection dose rate per gram of tissue (%ID/g) is expressed as mean ± standard deviation ($\bar{\chi }$ ± *s*). CB, contralateral bone; MB, model bone; CS, contralateral muscle.

**Table 5:** ROI ratio of model bone/contralateral bone in nude mice of bone metastasis model (*n* = 4).

ROI ratio	6 h	16 h	32 h
Mean	1.245	1.633	1.529
SD	0.295	0.472	0.187

## Data Availability

The data used to support the findings of this study are available from the corresponding author upon request.
